# Spindle component 25 predicts the prognosis and the immunotherapy response of cancers: a pan-cancer analysis

**DOI:** 10.1038/s41598-024-59038-y

**Published:** 2024-04-11

**Authors:** Fengjuan Xia, Haixia Yang, Huangjian Wu, Bo Zhao

**Affiliations:** 1 Department of Neurology of the First People’s Hospital of Zhaoqing, China, Zhaoqing, 526000 China; 2 Oncology Center of the First People’s Hospital of Zhaoqing, Zhaoqing, 526000 China; 3 Center for Pain Medicine of the First People’s Hospital of Zhaoqing, Zhaoqing, 526000 China

**Keywords:** Cancer, Computational biology and bioinformatics, Biomarkers, Oncology

## Abstract

Spindle component 25 (SPC25) is one of the four proteins that make up the nuclear division cycle 80 (NDC80) complex, the other three components being Ndc80p, Nuf2p, and spindle component 24. Deregulation of the components of this complex can lead to uncontrolled proliferation and reduced apoptosis. However, the prognostic and immunotherapeutic value of SPC25 in pan-cancer remains unclear. Data from the UCSC Xena, TIMER2.0, and TCGA were analyzed to investigate the overall differential expression of SPC25 across multiple cancer types. The survival prognosis, clinical features, and genetic changes of SPC25 were also evaluated. Finally, the relationship between SPC25 and immunotherapy response was further explored through Gene Set Enrichment Analysis, tumor microenvironment, and immune cell infiltration. The transcription and protein expression of SPC25 were significantly increased in most cancer types and had prognostic value for the survival of certain cancer patients such as ACC, CESC, KIRC, KIRP, LIHC, LUAD, MESO, STAD, THYM, and UCEC. In some cancer types, SPC25 expression was also markedly correlated with the TMB, MSI, and clinical characteristics. Gene Set Enrichment Analysis showed that SPC25 was significantly associated with immune-related pathways. In addition, it was also confirmed that the expression level of SPC25 was strongly correlated with immune cell infiltration, immune checkpoint genes, immune regulatory genes, Ferroptosis-related genes, Cuproptosis-related genes, and lactate metabolism-related genes. This study comprehensively explored the potential value of SPC25 as a prognostic and immunotherapeutic marker for pan-cancer, providing new direction and evidence for cancer therapy.

## Introduction

Undoubtedly, the global burden of cancer poses a monumental challenge to public health, obstructing the pursuit of enhanced longevity^1,2^. The realm of cancer treatment continues to grapple with grave circumstances. Tumors, notorious for their intricate biology, exhibit an array of processes—proliferation, evasion of growth inhibitors, resilience to cell demise, angiogenesis stimulation, invasion activation, and metastasis^3^. In addition, the dynamic interplay between tumor invasion and the host's immune response bears a striking correlation to the progression of cancer. As a critical player in tumor control, the immune system can facilitate efficacious immunotherapy by leveraging preexisting adaptive immune responses within the tumor, such as those involving checkpoint inhibitors^4^. In light of the widespread nature of tumors and the convoluted oncogenesis process, delving into the expression levels of pan-cancer-associated genes holds immense potential for advancements in clinical treatment and prognostic prediction.

The nuclear division cycle 80 (Ndc80) complex, vital to chromosome segregation, comprises four components: Spindle component 25 (SPC25), Ndc80p, Nuf2p, and Spindle component 24 (SPC24)^5^. Of these, SPC25 plays a pivotal role in orchestrating mitotic processes, forming heterodimers with SPC24 to regulate microtubule-centromeric attachment, chromosome organization, and spindle checkpoint activation^5,6^. Studies have revealed that aberrant chromosome segregation and compromised checkpoint signaling culminate in chromosomal instability (CIN)—a characteristic hallmark of numerous cancer types^7^. This phenomenon frequently arises during the initial stages of tumorigenesis, leading researchers to postulate that CIN may constitute the primary catalyst for tumor initiation^8,9^. Recent investigations have unveiled a noteworthy association between SPC25 dysregulation and carcinogenic processes, as well as malignant phenotypes in certain cancers. Moreover, in malignancies such as lung cancer, prostate cancer, and breast cancer, SPC25 expression is markedly elevated, thereby fueling tumor cell proliferation^10–15^. It is worth mentioning that SPC25 overexpression has been linked to unfavorable hepatocellular carcinoma (HCC) prognosis, as it fosters tumor growth and metastasis^16,17^.

In our investigation, we meticulously examined SPC25 expression across various cancer types, encompassing analyses of differentially expressed genes (DEGs), prognosis, and enrichment across diverse tumor classifications. Furthermore, we scrutinized the correlation between SPC25 expression, immune cell infiltration, and immunoregulatory factors. These findings not only imply that SPC25 could serve as a reliable prognostic biomarker, intricately connected to tumor immunomodulatory mechanisms, but also unveil its potential as a predictor for immunotherapy outcomes in the context of pan-cancer.

## Materials and methods

### Data collection

All data were obtained from the UCSC Xena database (https://genome.ucsc.edu/), where we downloaded gene expression data (FPKM was selected), mutation data, clinical data, and overall survival data from the GDC hub, and acquired other survival data from the Pan-Cancer Atlas Hub^18^. The specific sample numbers are as follows: Expression file:Thank you for your valuable comments. 33 tumor samples; Mutation Files: 33 tumor samples; Survival Files: 33 tumor samples. No data and values are missing.

### SPC25 differential expression analysis

TIMER2.0 database (Timer.cistrome.org) was used to analyze the differential expression levels of SPC25 mRNA in different cancer types and to demonstrate. Cancer and paracancer samples from the TCGA (https://portal.gdc.cancer.gov/) database were compared.

### Immunofluorescence and immunohistochemistry

The SPC25 distribution in cells and expression in cancer were reviewed by using the Human Protein Atlas (HPA, https://www.proteinatlas.org/). The SPC25 distribution in cells was examined by immunofluorescence, and the protein expression was examined by immunohistochemistry^19,20^.

### Survival analysis

The prognostic value of SPC25 was assessed using Kaplan–Meier analysis between high and low-expression groups. We performed univariate Cox regression analysis to examine the relationship between SPC25 expression and overall survival (OS), disease-related survival (DSS), and disease-free interval (DFI), after adjusting for age and tumor stage. *P*-value and hazard ratios (HR) with 95% confidence intervals (CI) were ascertained for each cancer type. Forest plots were generated using the R package “forestplot”^21,22^.

### Assessment of clinical correlations

Clinical correlation between SPC25 and pan-cancer was analyzed containing tumor stage (four stages) and age (defined by 65 years old) using the R-packages “limma” and “ggpubr”. *P*-value < 0.05 was considered statistically significant^23^.

### Gene set enrichment analysis

Pathways associated with SPC25 were investigated Using the “clusterprofiler” package. Statistical significance is indicated by an adjusted *p*-value < 0.05^24^.

### Correlation analysis of TMB and MSI with SPC25

Mutation data of SPC25 were all downloaded from the UCSC Xena database. The correlation of SPC25 with “Tumor Mutation Burden” (TMB) and “Microsatellite Instability” (MSI) of each cancer was tested using the “Spearman” method, shown in the radar plots. The TMB score for each sample was calculated using the TCGA pan-cancer mutation data, while the MSI score was obtained from a previous study^25^.

### Tumor microenvironment and immune infiltrate analysis

To analyze the infiltration levels of immune cells and stromal cells in pan-cancer, we used ESTIMATE to determine the correlation between SPC25 expression and immune score, stromal score. Moreover, the relationship between SPC25 expression level and different immune cells (CD8 T cells and monocytes, etc.) was analyzed using the CIBERSORT method (http://cibersort.stanford.edu/) and TIMER2.0 database (http://timer.cistrome.org/). The *p* filter was set to less than 0.001 ^26–28^.

### Co-expression analysis of the SPC25 gene

At the same time, the correlation between SPC25 and other genes was evaluated. These genes contained Cuproptosis-related genes, Ferroptosis-related genes, lactate metabolism-related genes, and immune chemokines genes. Heatmaps were used to show the results of the co-expression analyses.

Co-expression gene set source:

1. lactate metabolism related genes

Establishment of lactate-metabolism-related signature to predict prognosis and immunotherapy response in patients with colon adenocarcinoma.

(2) Immune chemokines genes

Pan-cancer integrated bioinformatics analysis reveals cuproptosis related gene FDX1 is a potential prognostic and immunotherapeutic biomarker for lower-grade gliomas.

(3) Cuproptosis-related genes

Cuproptosis correlates with immunosuppressive tumor microenvironment based on pan-cancer multiomics and single-cell sequencing analysis.

(4) Ferroptosis-related genes

Farnesoid X receptor protects against cisplatin-induced acute kidney injury by regulating the transcription of ferroptosis-related genes; Identification and verification of ferroptosis-related genes in the synovial tissue of osteoarthritis using bioinformatics analysis.

Instructions:

Intersection co-expression analysis of SPC25 gene expression and target gene sets in 33 tumors using the limma package in R-4.2.1.

### Cell culture

NHAS, U87, U373, BT325 and RSHG-44 cells were purchased from the CAS Shanghai Cell Bank. The cell culture medium was DMEM (Gibco), containing 10% serum (FBS, Gibco) and antibiotics. Place the cells in a 37 °C culture chamber filled with 5% carbon dioxide.

### Western blot

Harvest cells with RIPA lysate and perform BCA determination of protein concentration. Then, SDS-PAGE is performed, and the proteins are transferred to the PVDF membrane. After 1 h of blocking with 5% BSA at room temperature, incubate the membrane with the primary antibody overnight and then with the secondary antibody for 1 h. Finally, the signal is detected using an ECL kit.

### Statistical analysis

All statistical analyses were performed by the R (https://www.r-project.org/). *p* < 0.05 was considered statistically significant, and we have marked * in the results of the different analyses, where * represents *p* < 0.05, ** represents *p* < 0.01, and *** represent *p* < 0.001. R language version: R-4.2.1.

## Result

### Analysis of SPC25 expression in pan-cancers

**Figure 1:**
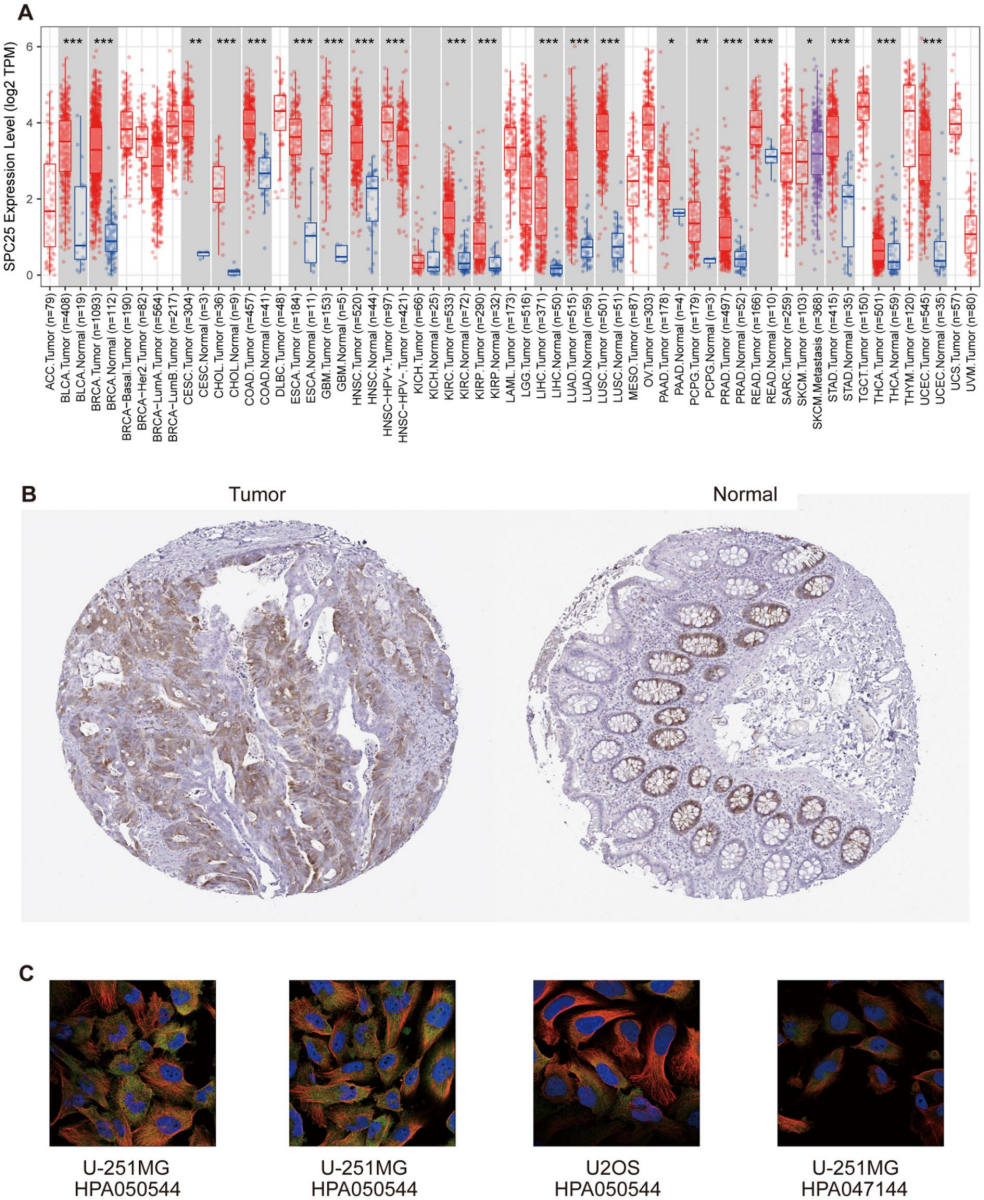
Differences in SPC25 expression between normal and tumor tissue samples (**A**) expression of SPC25 in pan-cancer and corresponding normal samples in TIMER2.0 (**B**) normal colon tissue and colon cancer (**C**) SPC25 cell location, green indicates the distribution of SPC25. The red box represents the tumor (T) and the blue box represents normal tissue (N).

The differential analysis of SPC25 based on cancer and pan-cancer tissue samples from the TCGA and TIMER2.0 database indicated that SPC25 had higher expression in cancer tissues than paraneoplastic tissues such as BLCA BRCA, CESC, CHOL, COAD, ESCA, GBM, HNSC, KIRC, KIRP, LIHC, LUAD, LUSC, PRAD, READ, STAD, THCA, and UCEC (Fig. 1A). For cancers lacking pan-cancer tissue, we found no significant difference. Through the HPA database, we found that the expression level of commercial SPC25 in Colon adenocarcinoma (COAD) was higher than that in normal tissues (Fig. 1B). Moreover, we also determined that SPC25 is mainly located in the cytoplasm. Cell localization of SPC25 was obtained in U-251MG and U2OS cells using antibodies HPA050544 and HPA047144. (Fig. 1C).

### Prognostic value of SPC25 in cancer patients

**Figure 2:**
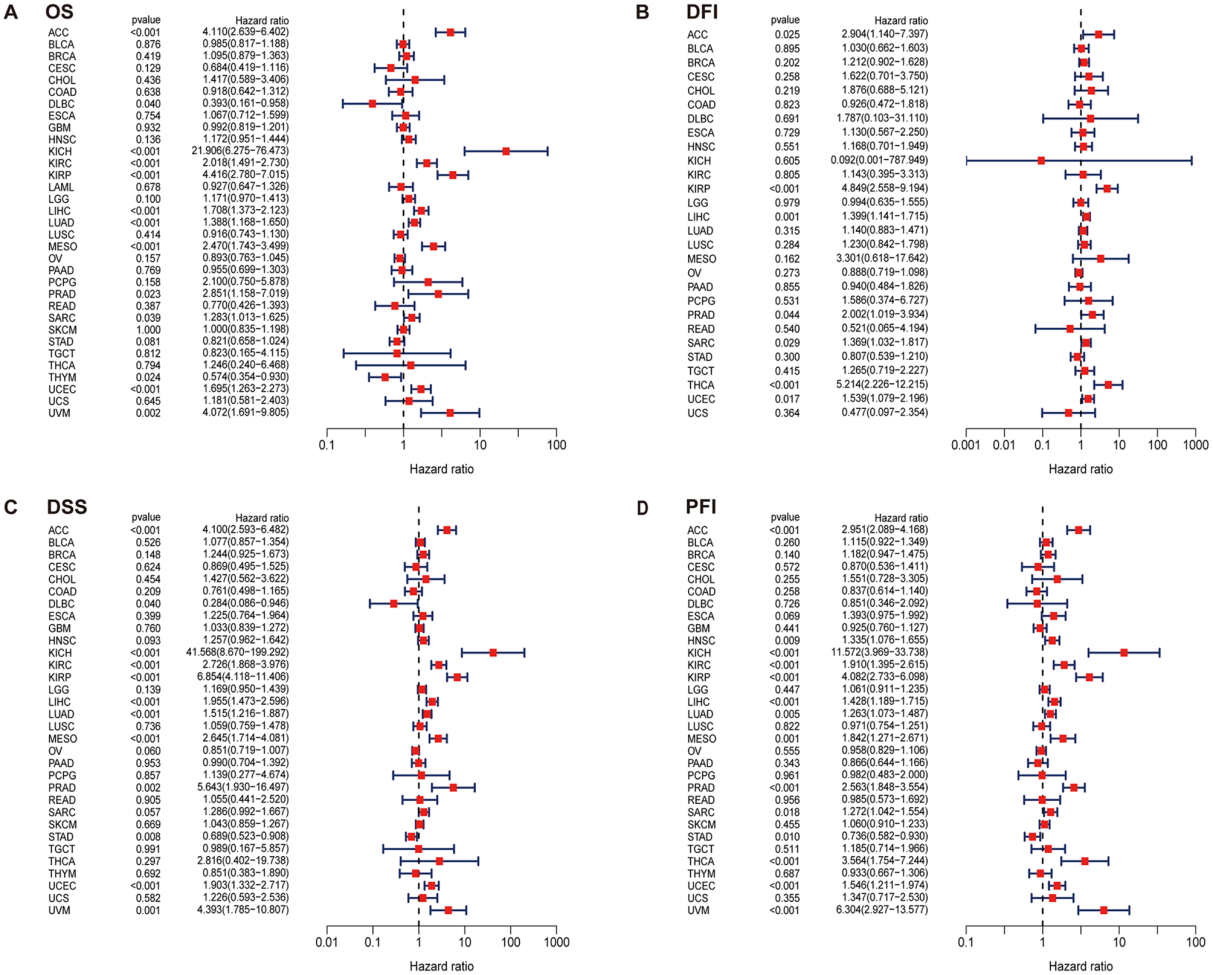
Prognostic assessment of SPC25 expression in OS, DFI, DSS and PFI. Correlation between SPC25 expression and OS (**A**) DFI (**B**) DSS (**C**) PFI (**D**) by utilizing Cox. HR (hazard ratio) > 1 indicates that SPC25 may be an adverse factor in the occurrence and development of cancer; 0 < HR < 1 indicates that SPC25 may be a protective factor in cancer.

The potential prognostic value of SPC25 was assessed using Cox proportional hazards model and Kaplan Meier analysis. The results of the Cox model showed that the expression level of SPC25 was positively associated with the prognosis of ACC (*p* < 0.001), KICH (*p* < 0.001), KIRC (*p* < 0.001), KIRP (*p* < 0.001), LIHC (*p* < 0.001), LUAD (*p* < 0.001), MESO (*p* < 0.001), and UCEC (*p* < 0.001), as well as negatively in THYM (*p* = 0.024) and DLBC (*p* = 0.040). Forest plots showed that high expression of SPC25 predicted poor DFI in KIRP (*p* < 0.001), and THCA (*p* < 0.001). For DSS, high expression of SPC25 was a negative factor in ACC (*p* < 0.001), KICH (*p* < 0.001), KIRC (*p* < 0.001), KIRP (*p* < 0.001), LIHC (*p* < 0.001), LUAD (*p* < 0.001), MESO (*p* < 0.001), PARAD (*p* < 0.001), and UCEC (*p* < 0.001) patients, but a positive factor in DLBC patients (*p* = 0.040) and STAD (*p* = 0.008). Furthermore, in the PFI-related Cox proportional hazards model, SPC25 also showed significant prognostic value in ACC (*p* < 0.001), KICH (*p* < 0.001), KIRC (*p* < 0.001), KIRP (*p* < 0.001), LIHC (*p* < 0.001), PRAD (*p* < 0.001), THCA (*p* < 0.001), UCEC (*p* < 0.001), and UVM (*p* < 0.001) (Fig. 2).

**Figure 3:**
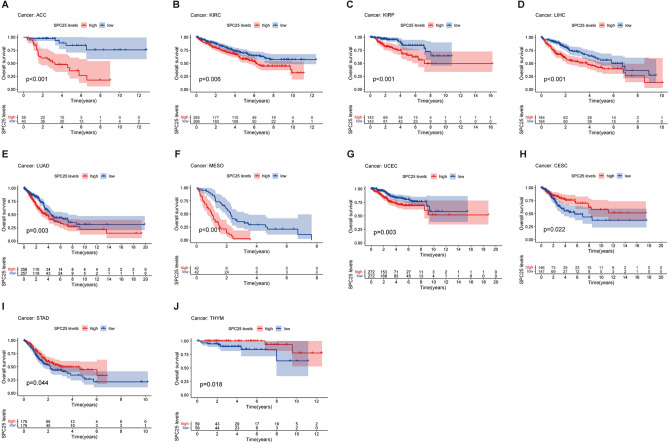
Kaplan–Meier analysis of OS in patients with high and low SPC25 expression (**A**–**G**) high expression of SPC25 predicted poor OS than low expression (**H**–**J**) high expression of SPC25 predicted better OS than low expression.

Kaplan–Meier analysis showed that high expression of SPC25 predicted poor OS in ACC (*p* < 0.001), KIRC (*p* = 0.006), KIRP (*p* < 0.001), LIHC (*p* < 0.001), LUAD (*p* = 0.003), MESO (*p* < 0.001), and UCEC (*p* = 0.003), while high SPC25 predicted better OS in THYM (Fig. 2D , *p*= 0.018), CESC (*p* = 0.022), STAD (*p* = 0.044), and THYM (*p* = 0.018) (Fig. 3).

About DFI, Kaplan–Meier analysis found that LIHC is statistically significant (*p* = *p* = 0.004). In addition, the K-M curve of KIRP (*p* = 0.016), UCEC (*p* = 0.049), and THCA (*p* = 0.015) showed that high expression of SPC25 indicated poor prognosis. While in STAD (*p* = 0.017), high expression of SPC25 indicated a good prognosis (Supplementary Fig. 2).

Consistent with the results of the Cox proportional hazards model of DSS, the K-M curve indicated that a high level of SPC25 was positively correlated with good survival outcomes in STAD (*p* = 0.002), and COAD (*p* = 0.031), and negatively correlated with survival in ACC (*p* < 0.001), KIRP (*p* < 0.001), LUAD (*p* < 0.001), MESO (*p* < 0.001), LIHC (*p* = 0.003), UCEC (*p* = 0.002), and KIRC (*p* = 0.003) (Supplementary Fig. 3).

Finally, Kaplan–Meier analysis also showed that high SPC25 expression predicted poor PFI in ACC (*p* < 0.001), KIRP (*p* < 0.001), LUAD (*p* = 0.003), LIHC (*p* < 0.001), MESO (*p* < 0.001), PRAD (*p* < 0.001), UCEC (*p* = 0.001) and UVM (*p* < 0.001), then showed better PFI in COAD (*p* = 0.045) and STAD (*p* < 0.001) (Supplementary Fig. 5).

### The relationship between SPC25 and clinical information

**Figure 4:**
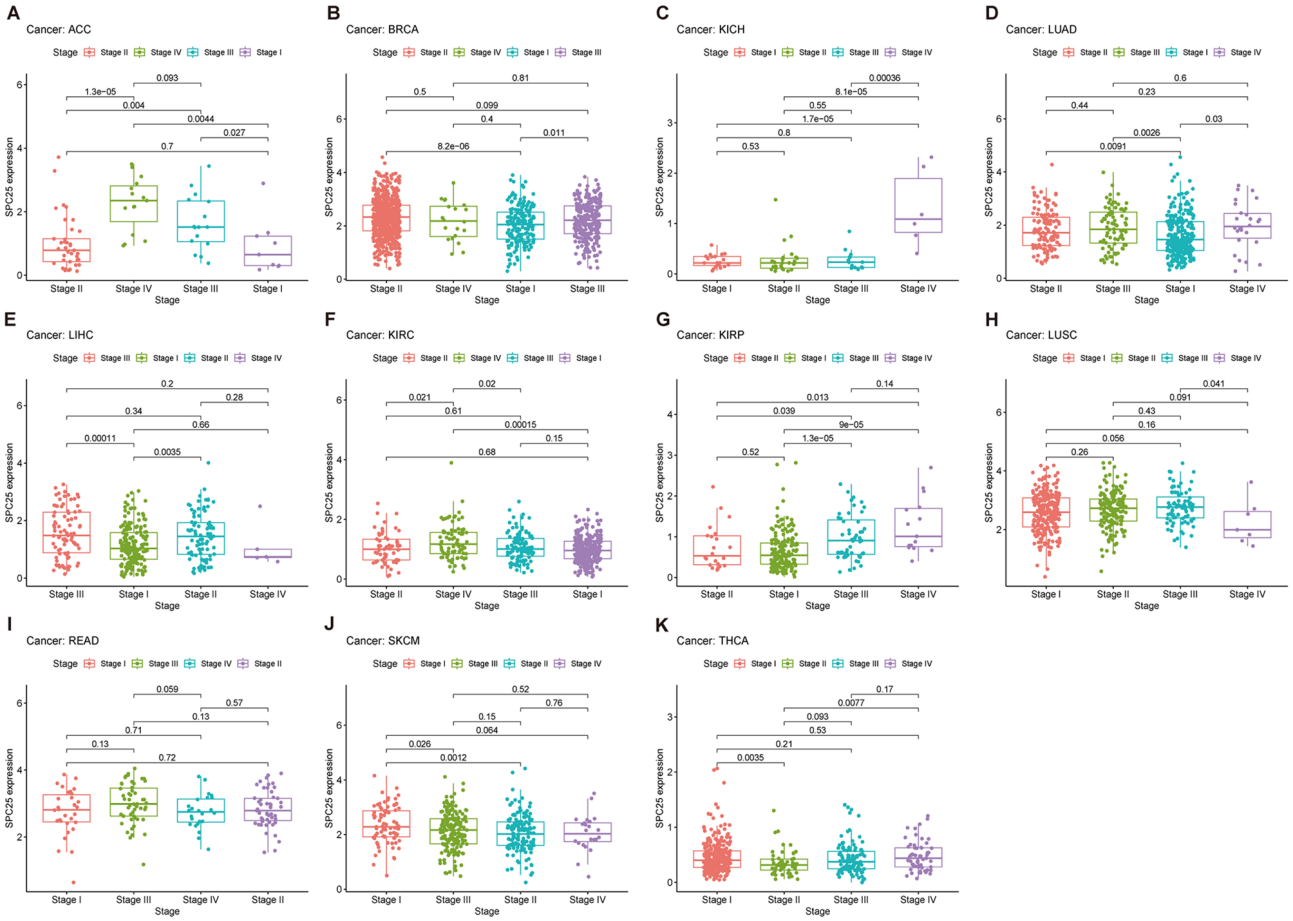
Analysis of clinic correlation of SPC25 expression. Correlation analysis of SPC25 expression and tumor clinical stage (**A**) ACC (**B**) BRCA (**C**) KICH (**D**) LUAD (**E**) LIHC (**F**) KIRC (**G**) KIRP (**H**) LUSC (**I**) READ (**J**) SKCM (**K**) THCA.

In the advanced stages of ACC and KIRP, especially in stage III and stage IV, the expression of SPC25 was significantly higher than early stages. In KICH, the expression of SPC25 was the highest in stage IV, which was markedly different from stages I, II, and III. It might be linked to excessive proliferation, poor prognosis, invasion of stage IV cancer cells, and cell death inhibition. In stage II, stage III, and stage IV of LUAD and KIRC, SPC25 was higher compared to stage I. However, the sample size of stage IV LUAD was too small to effectively indicate the comparative results. Furthermore, the expression of SPC25 was higher in stages I of THCA and SKCM. SPC25 expression in ESCA, KIRP, and LAML was significantly higher in patients under 65 years of age than in patients over 65 years of age. In contrast, SPC25 expression in STAD, LUSC, and THYM was significantly higher in patients older than 65 years than in patients younger than 65 years (Fig. 4, Supplementary Fig. 5).

### Relationship of SPC25 with TMB and MSI

**Figure 5:**
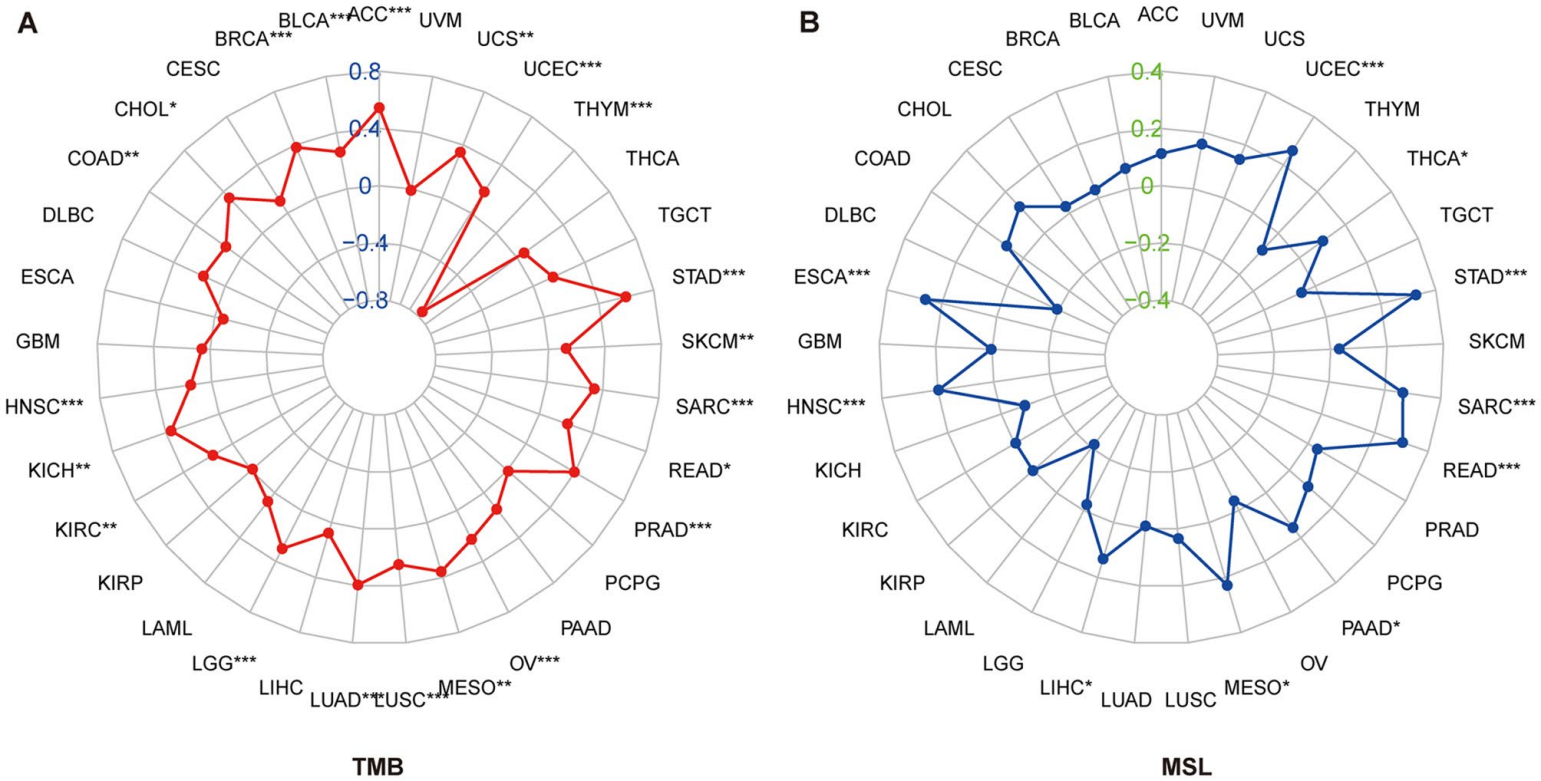
Relationship of SPC25 expression with TMB (**A**) and MSI (**B**).

More and more studies show that TMB and MSI may be independent biomarkers reflecting the therapeutic effect of immune checkpoint inhibitors and the prognosis of cancers^29,30^. Thence, we further explored the relationship of SPC25 with TMB and MSI in the pan-cancer cohort. SPC25 expression was positively related to the TMB in ACC, UCS, UCEC, STAD, SKCM, SARC, PRAD, OV, MESO, LUSC, LUAD, LGG, KIRC, KICH, HNSC, BRCA, and BLCA, whereas the negative association was observed in THYM. For MSI, a positive association was obtained in UCEC, STAD, SARC, READ, ESCA, and HNSC, the negative association was shown in DLBC, THYM, LAML, and KICH (Fig. 5).

### GSEA analysis of SPC25

**Figure 6:**
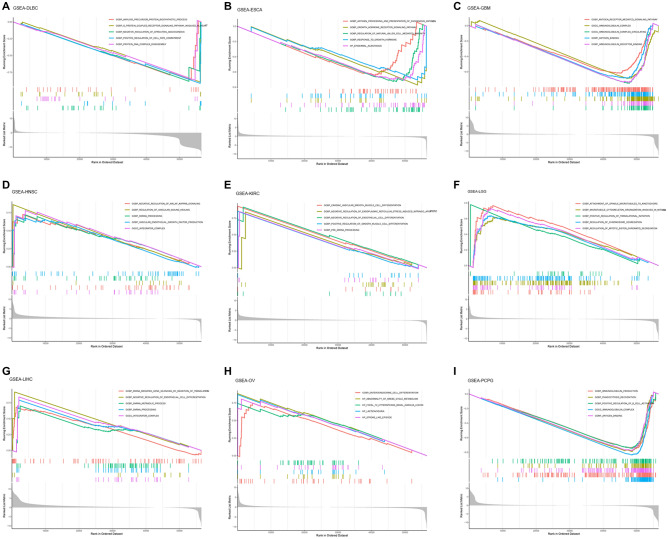
Gene set enrichment analysis of SPC25 in Pan-cancer. (**A**) DLBC (**B**) ESCA (**C**) GBM (**D**) HNSC (**E**) KIRC (**F**) LGG (**G**) LIHC (**H**) OV (**I**) PCPG.

Single-gene GSEA was used to identify relevant pathways affected by SPC25 expression in pan-cancer. The results showed that SPC25 is positively correlated with the immune-related pathways in ESCA, THCA, PCPG, and GBM. SPC25 is positively correlated with HNSC, KIRC, LGG, LIHC, and OV signal pathways, such as cell division, spindle filament activity, and smooth muscle cell activity. Also, SPC25 was positively correlated with the chromosome activity, nuclear organization, and cell metastasis of PRAD, STAD, and THYM (Supplementary Fig. 1). Furthermore, we observed that SPC25 is negatively correlated with cell metabolism, NK cell activity, and vascular regulation in DLBC, GBM, ESCA, SARC, and PCPG, and negatively correlated with READ and ECEC signal pathways, such as axoneme activity, vascular-associated smooth muscle activity, and zymogen activation. Our findings suggested that SPC25 generally correlated with many important pathways in cancer formation (Fig. 6).

### Correlation between SPC25 expression and immune infiltrating level in pan-cancers

**Figure 7:**
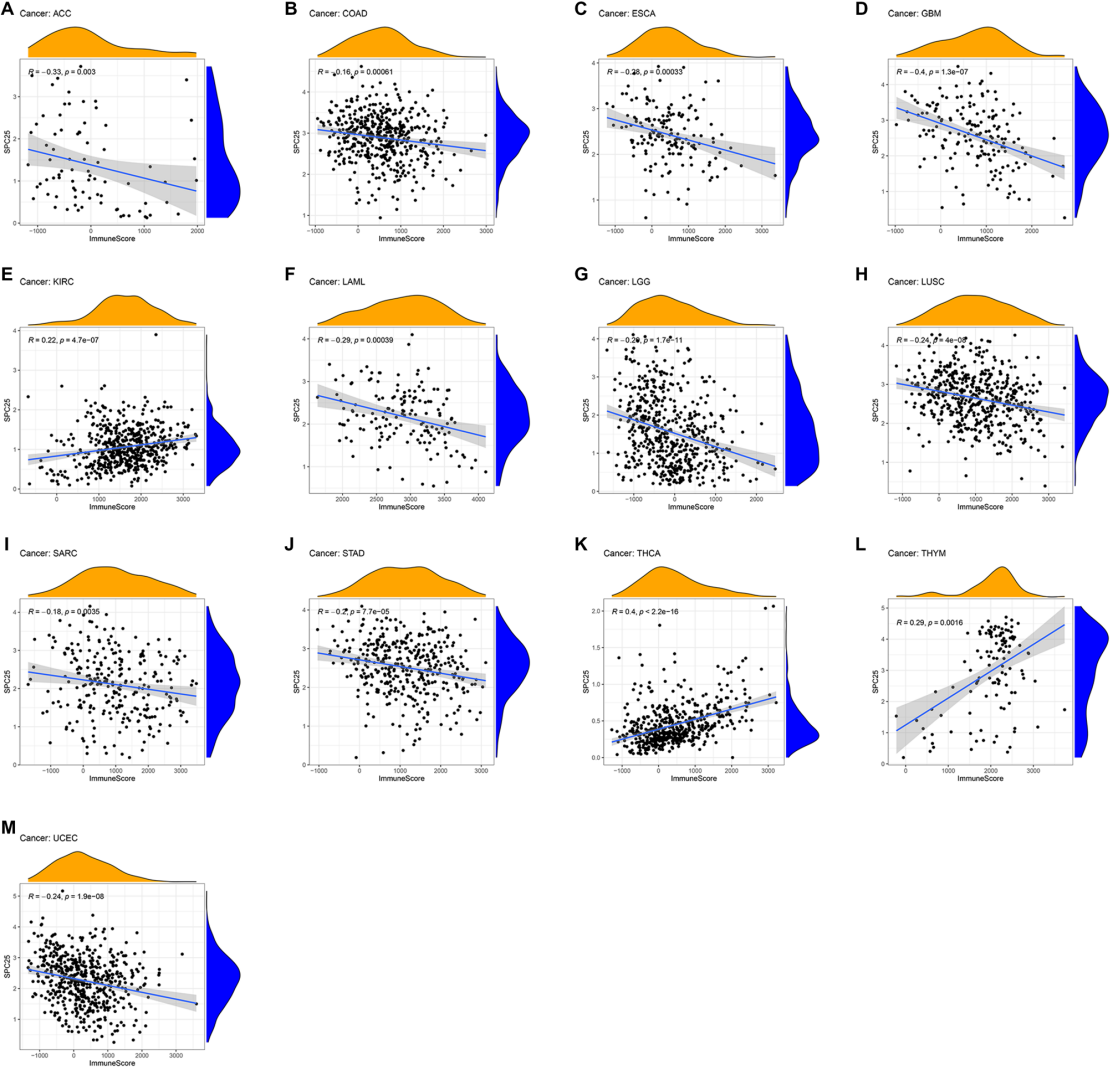
Association of SPC25 with the TME Composition. Immune Score (**A**) ACC (**B**) COAD (**C**) ESCA (**D**) GBM (**E)** KIRC (**F**) LAML (**G**) LGG (**H**) LUSC (**I**) SARC (**J**) STAD (**K**) THCA (**L**) THYM (**M**) UCEC.

**Figure 8:**
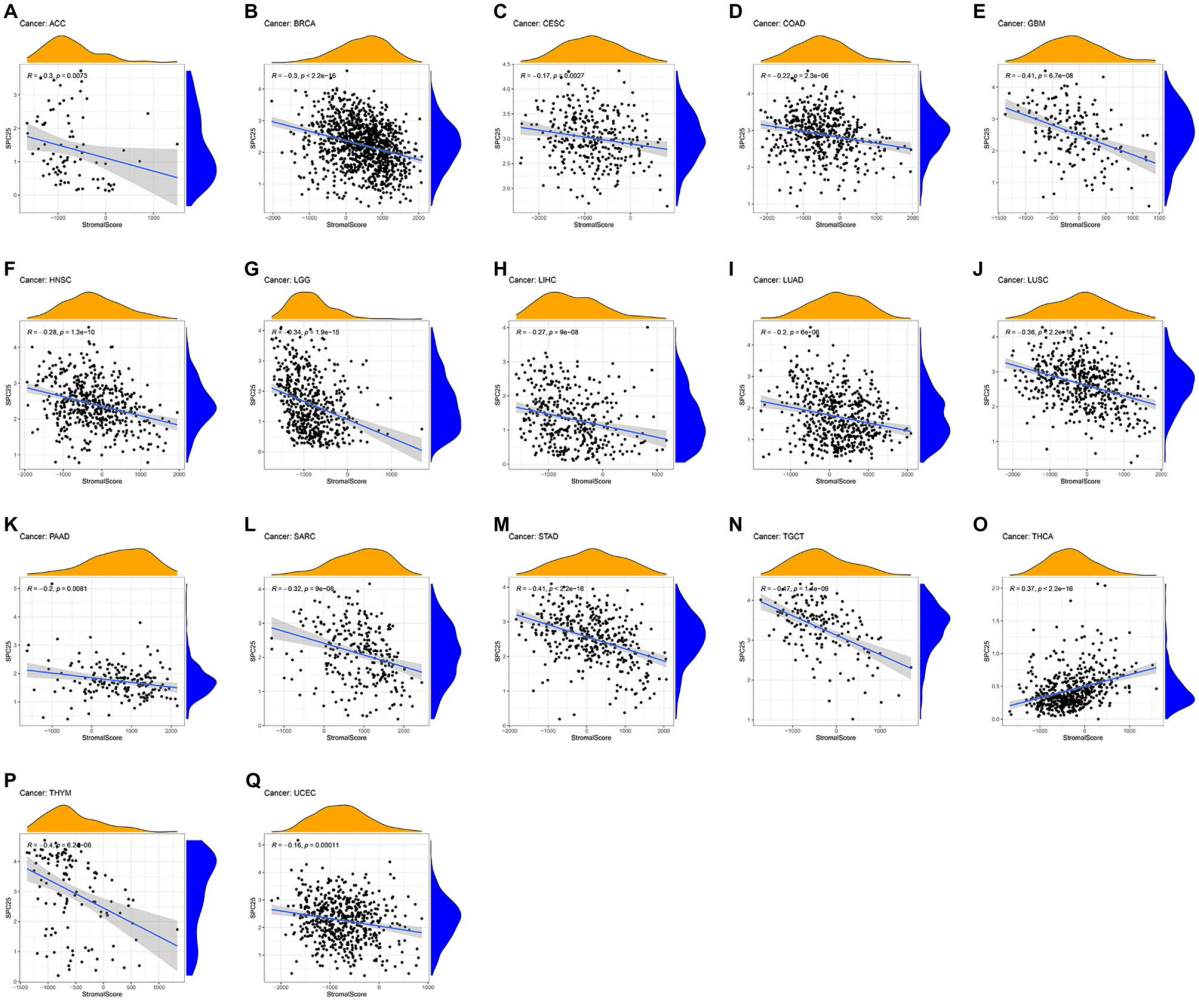
Association of SPC25 with the TME Composition. StromalScore (**A**) ACC (**B**) BRCA (**C**) CESC (**D**) COAD (**E**) GBM (**F**) HNSC (**G**) LGG (**H**) LIHC (**I**) LUAD (**J**) LUSC (**K**) PAAD (**L**) SARC (**M**) STAD (**N**) TGCT (**O**) THCA (**P**) THYM (**Q**) UCEC.

According to GSEA, we observed a potential association between SPC25 and immune-related factors. Therefore, we further performed tumor microenvironment and immune infiltrate analysis. The results showed that SPC25 had a positive correlation with the immune score in KIRC (R = 0.22), THYM (R = 0.29), and THCA (R = 0.4), while SPC25 was a negative correlation with the immune score in ACC (R = − 0.33), COAD (R = − 0.16), ESCA (R = − 0.28), GBM (R = − 0.4), LAML (R = − 0.29), LGG (R = − 0.20), LUSC (R = − 0.24), SARC (R = − 0.18), STAD (R = − 0.2), and UCEC (R = − 0.24) (Fig. 7). For stromal scores, positive correction with SPC25 was identified in THCA (R = 0.37). SPC25 was negatively correlated with the stromal score of ACC (R =  − 0.3), BRAC (R =  − 0.3), CESC (R =  − 0.17), COAD (R =  − 0.22), GBM (R =  − 0.41), HNSC (R =  − 0.28), LGG (R =  − 0.34), LIHC (R =  − 0.27), LUAD (R =  − 0.2), LUSC (R =  − 0.36), PAAD (R =  − 0.2), SARC (R =  − 0.32), STAD (R =  − 0.41), TGCT (R =  − 0.47), THYM (R =  − 0.4), and UCEC (R =  − 0.16). In ACC (immune scores: R =  − 0.33, stromal score: R =  − 0.3), COAD (immune scores: R =  − 0.16, stromal score: R =  − 0.22), GBM (immune scores: R =  − 0.4, stromal score: R =  − 0.41), LGG (immune scores: R =  − 0.20, stromal score: R =  − 0.36), LISC (immune scores: R =  − 0.24, stromal score: R =  − 0.41), SARC (immune scores: R =  − 0.18, stromal score: R =  − 0.32), STAD (immune scores: R =  − 0.2, stromal score: R =  − 0.41), THCA (immune scores: R = 0.4, stromal score: R = 0.37), THYM (immune scores: R = 0.29, stromal score: R =  − 0.4), and UCEC (immune scores: R =  − 0.24, stromal score: R =  − 0.16), SPC25 transcript levels were consistently negatively correlated with immune and stromal scores (Fig. 8).

**Figure 9:**
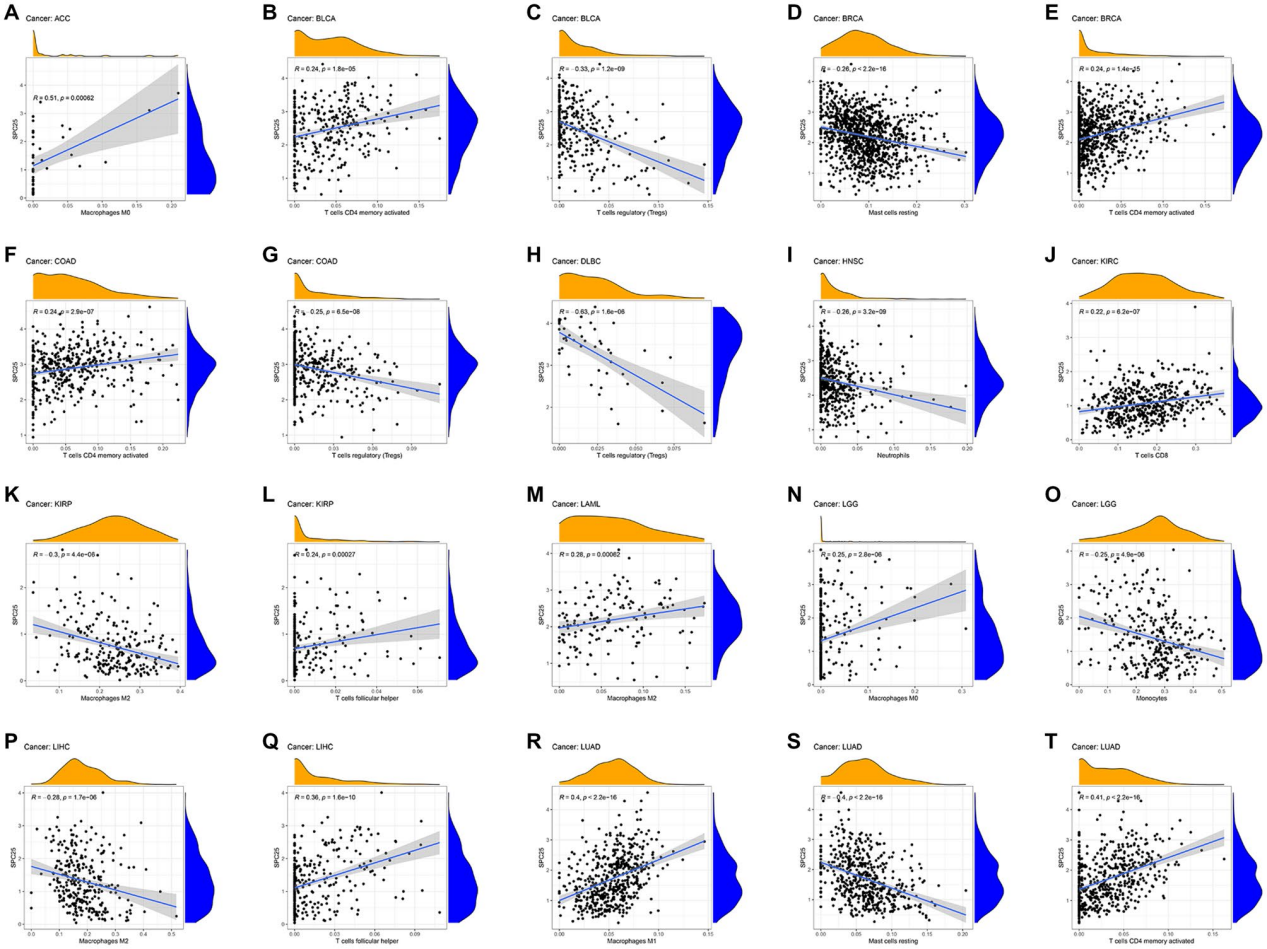
The correlation between SPC25 expression and immune cell infiltration (**A**–**T**) Scatter plots showing that tumor-infiltrating immune cells were significantly correlated with SPC25 expression.

Immune-related cell infiltration is the main mechanism affecting the tumor microenvironment. Therefore, we further studied the relationship between SPC25 expression and immune infiltration analysis in pan-cancer. We found that SPC25 was positively correlated with macrophages in ACC, LAML, LGG, and LUAD, while was negatively correlated with KIRP, and LIHC. On the other hand, SPC25 showed a correlation with T cells in BLCA, BRCA, COAD, DLBC, KIRC, and KIRP. In addition, SPC25 is negatively correlated with mast cells in BRCA and neutrophils in HNSC (Fig. 9).

### SPC25 correlated with the majority of Cuproptosis-related genes, Ferroptosis-related genes, lactate metabolism-related genes, and immune chemokines genes

In conclusion, SPC25 may have a significant relationship with tumor immune regulation, so we further explored the role of SPC25 at the gene level. We performed comprehensive co-expression analysis on the Cuproptosis-related genes, Ferroptosis-related genes, lactate metabolism-related genes, and immune chemokines genes. The results showed that immune chemokines genes 47 immune checkpoint genes showed that most genes in LIHC, THCA, and THYM were significantly positively correlated with SPC25, while negatively correlated in GBM, LGG, PRAD, STAD, and UCEC. The results showed that Ferroptosis-related genes such as SLC3A2, HSF1, and SLC7A11 were significantly positively correlated with SPC25 in pan-cancer. In Cuproptosis-related genes, SPC25 was positively correlated with GCSH, CDKN2A, PDHB, PDHA1, DLAT, DLD, and SLC31A1 of pan-cancer. TWNK, TRMU, RNASEH1, PIF1, PDSS1, MGME1, DNA2, and DARS2 in lactate metabolism-related genes were significantly correlated with the expression of SPC25 in pan-cancer. Similarly, we can see that most of the lactate metabolism-related genes in BLCA, BRCA, ESCA, HNSC, LGG, LIHC, SKCM, and UVM are closely related to the expression of SPC25 (Supplementary Fig. 6).

## Discussion

Through an exhaustive examination, our research illuminated the prognostic and potential immunotherapeutic significance of SPC25 in the context of pan-carcinoma. We observed markedly elevated gene levels of SPC25 in the majority of tumors, with some cancers demonstrating distinct prognostic implications. Moreover, the expression of SPC25 bears a strong connection to immune and inflammatory pathways, immune cell infiltration, and an array of immune-associated genes. Consequently, SPC25 emerges as a potential prognostic biomarker and a predictor of immunotherapy outcomes.

SPC25, an integral component of the NDC80 complex, plays a pivotal role in assembling the microtubule-binding domain of kinetochores and facilitating chromosome alignment with the metaphase plate. In concert with SPC24, SPC25 orchestrates chromosome alignment and spindle formation, processes indispensable for chromosome segregation^31^. As a crucial element, it auto-inhibits the interaction between the microtubule and the Ndc80 complex, thereby regulating chromosome segregation during cell mitosis—a critical process intertwined with tumorigenesis^32,33^. Hitherto, research on SPC25 has primarily centered on its role in cell mitosis, where dysregulation has been implicated in abnormal cell division. Data concerning the relationship between SPC25 and cancer development remain scarce. Nonetheless, statistical analyses drawing upon online databases, such as TCGA, insinuate a close association between SPC25 expression and poor prognosis in patients with hepatocellular carcinoma (HCC), non-small cell lung adenocarcinoma cells, and prostate cancer. Preliminary in vitro and in vivo functional tests of tumor cells have corroborated that elevated SPC25 expression can stimulate HCC cell proliferation and augment cancer stem cell (CSC) characteristics^14,34^. In light of this evidence, our focus gravitates toward the mechanisms underpinning SPC25's involvement in multiple tumors, delving deeper at the pan-cancer level.

Upon evaluating SPC25 mRNA levels in 33 human tumors across multiple databases, we discerned a significant reduction in expression across the majority of tumors. Contrasting transcription levels, we found that BLCA, BRCA, CESC, CHOL, COAD, ESCA, GBM, HNSC, KIRC, KIRP, LIHC, LUAD, LUSC, PRAD, READ, STAD, THCA, and UCEC exhibited markedly elevated transcription levels. For most cancers, SPC25 expression exhibited no significant discrepancies based on clinical stage, age, or sex. However, in certain cancers, particularly ACC, KICH, KIRC, KIRP, LIHC, MESO, UCEC, and UVM, SPC25 expression emerged as an independent prognostic factor and a potential prognostic marker.

Furthermore, GSEA enrichment findings revealed that SPC25 exhibited a strong association with immune and inflammatory responses, protein metabolism, spindle filament activity, and chromosome activity, aligning with established tumor mechanisms. We honed in on various immune pathways, encompassing inflammatory responses, T-cell reactions, and macrophage response signaling pathways, which are intimately linked to tumorigenesis. To delve deeper into SPC25's potential value, we probed its relationship with the tumor microenvironment and immune cell infiltration. Our results uncovered a robust correlation between SPC25 and the infiltration of diverse immune cell types (such as CD4 + T cells, macrophages, and T cell regulation) within multiple tumor TMEs. Consequently, we surmise that SPC25 may modulate immune cells in the tumor microenvironment via numerous pathways, as opposed to exclusively targeting specific immune cells.

Ordinarily, the immune system employs immune cell infiltration to identify and eradicate tumor cells within the TME. The infiltration of immune cells and the elicitation of anti-tumor immune responses are chiefly governed by an array of chemokines, chemokine receptors, cytokines, and immune checkpoints. Bearing these factors in mind, we assessed the connection between SPC25 and immune checkpoint genes. Intriguingly, SPC25 exhibited a significant association with the majority of genes in BLCA, KIRC, LIHC, THCA, and THYM malignancies, aligning closely with prior GSEA enrichment outcomes. This intimates that SPC25 could function as a potential regulatory target in the immunotherapy of these cancers and suggests its involvement in inducing immune cell recruitment is far from trivial^35–37^.

The tumor immune microenvironment plays an important role in tumor development, prognosis, and immunotherapy^38,39^. In ACC and COAD, SPC25 was significantly negatively correlated with immune score and immune cell infiltration. However, different immune cell types also play different roles in cancer, for example, CD8 + T cells tend to be associated with a good prognosis, while regulatory T cells are mostly associated with a poor prognosis^4^. Immune cell infiltration analysis showed a significant negative correlation between SPC25 and regulatory T-cell infiltration, suggesting that COAD patients with high SPC25 expression may have a better prognosis, which is also consistent with our analysis results. COAD is a rare but highly malignant tumor, and treatment options for advanced cancers are severely limited. Therefore, immunotherapies such as checkpoint inhibitors and monoclonal antibodies may eventually be accepted as effective potential treatments for these patients^40^. Importantly, SPC25 showed a significant positive correlation with COAD expressing many immune checkpoint genes, especially NRP1, CD276, TNFSF14, and other important immunotherapeutic targets, which may also be closely related to the immune escape mechanism of ACC tumor cells.

## Conclusion

We found that SPC25 is a novel biomarker for a variety of cancers. We found that SPC25 was significantly associated with prognosis, mutation, and immunity in pan-cancer. It promises to be a new therapeutic target for many types of cancer.

### Supplementary Information

Supplementary Legends.

Supplementary Figure 1.

Supplementary Figure 2.

Supplementary Figure 3.

Supplementary Figure 4.

Supplementary Figure 5.

Supplementary Figure 6.

## Data Availability

The original contributions presented in the study are included in the article/Supplementary Materials, further inquiries can be directed to the corresponding author.
