# Supplementary material for: Spindle component 25 predicts the prognosis and the immunotherapy response of cancers: a pan-cancer analysis
Source: Sci Rep. 2024 Apr 11;14:8452. doi: 10.1038/s41598-024-59038-y (PMC11009294; doi:10.1038/s41598-024-59038-y)
Supplement: Supplementary file 1 — Supplementary Legends. [file 41598_2024_59038_MOESM1_ESM.docx]

Supplementary Figure1

Single-gene GSEA of SPC25.

Supplementary Figure2

Kaplan-Meier analysis of DFI in patients with high and low SPC25 expression. (A-D) high expression of SPC25 predicted poor DFI than low expression (E) high expression of SPC25 predicted better DFI than low expression.

Supplementary Figure3

Kaplan-Meier analysis of DSS in patients with high and low SPC25 expression. (A-G) high expression of SPC25 predicted poor DSS than low expression (H-I) high expression of SPC25 predicted better DSS than low expression.

Supplementary Figure4

Kaplan-Meier analysis of PFI in patients with high and low SPC25 expression. (A-H) high expression of SPC25 predicted poor PFI than low expression (I-J) high expression of SPC25 predicted better PFI than low expression.

Supplementary Figure5

Analysis of clinic correlation of SPC25 expression. Correlation analysis of SPC25 expression and age (with 65 years as the cutoff) (A) ESCA (B) KIRP (C) LAML (D) STAD (E) LUSC (F) THYM. Red means the patient is younger than 65 years old, and blue means the patient is 65 years or older.

Supplementary Figure6

Gene co-expression analysis of SPC25 in pan-cancer. The heatmaps presenting the correlations of SPC25 expression with genes related to (A) immune chemokines genes (B) Ferroptosis-related genes (C) Cuproptosis-related genes and (D) lactate metabolism related genes. The upper left corner of each square represents the p-value, * represents p < 0.05, ** represent p < 0.01, and *** represent p < 0.001; the lower right corner represents the correlation between SPC25 and other genes, red represents positive correlation, and yellow represents negative correlation.
