# Supplementary figures and images for: Spindle component 25 predicts the prognosis and the immunotherapy response of cancers: a pan-cancer analysis
Source: Sci Rep. 2024 Apr 11;14:8452. doi: 10.1038/s41598-024-59038-y (PMC11009294; doi:10.1038/s41598-024-59038-y)

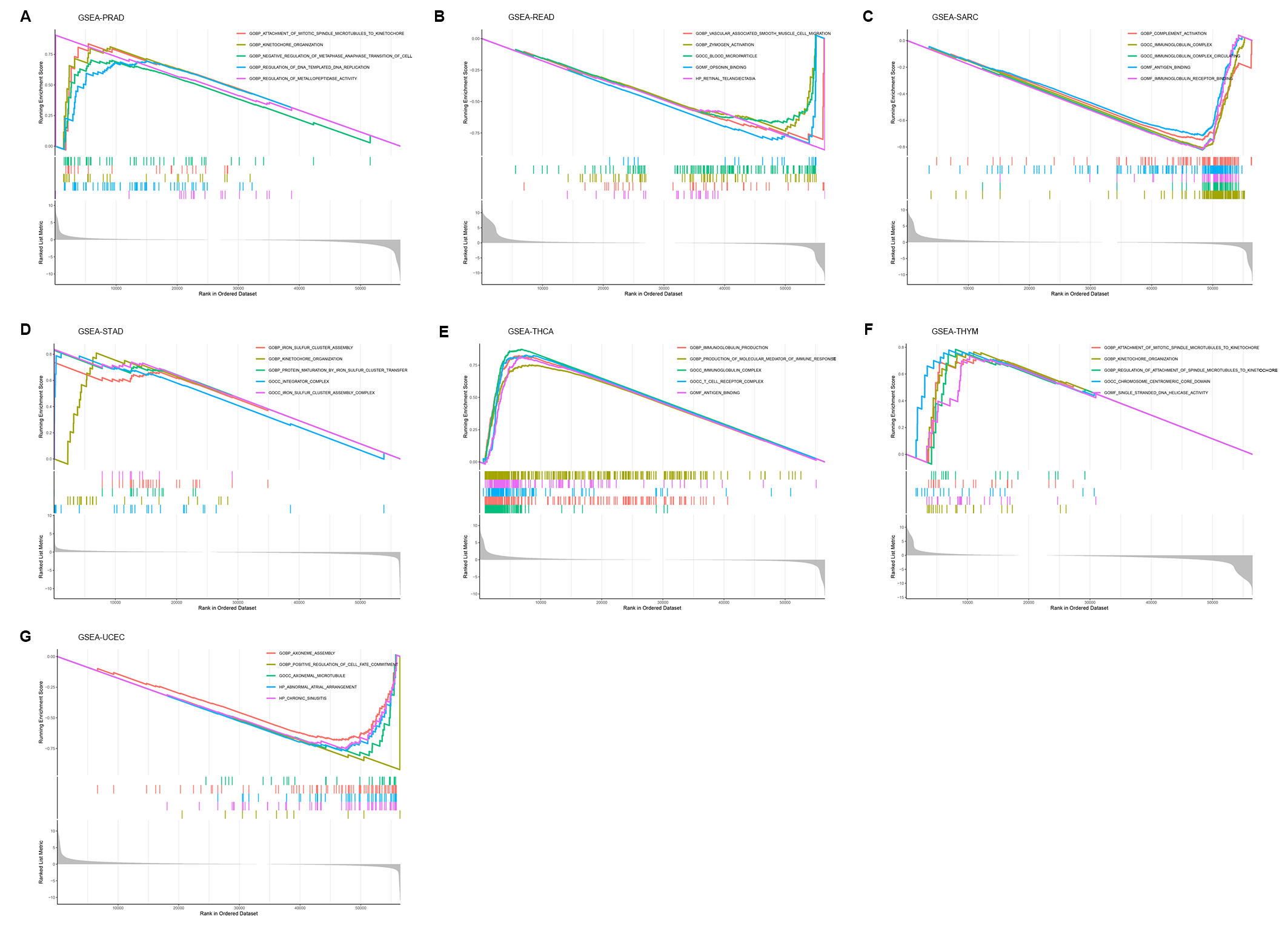

Supplement: Supplementary file 2 — Supplementary Figure 1. [file 41598_2024_59038_MOESM2_ESM.tif]

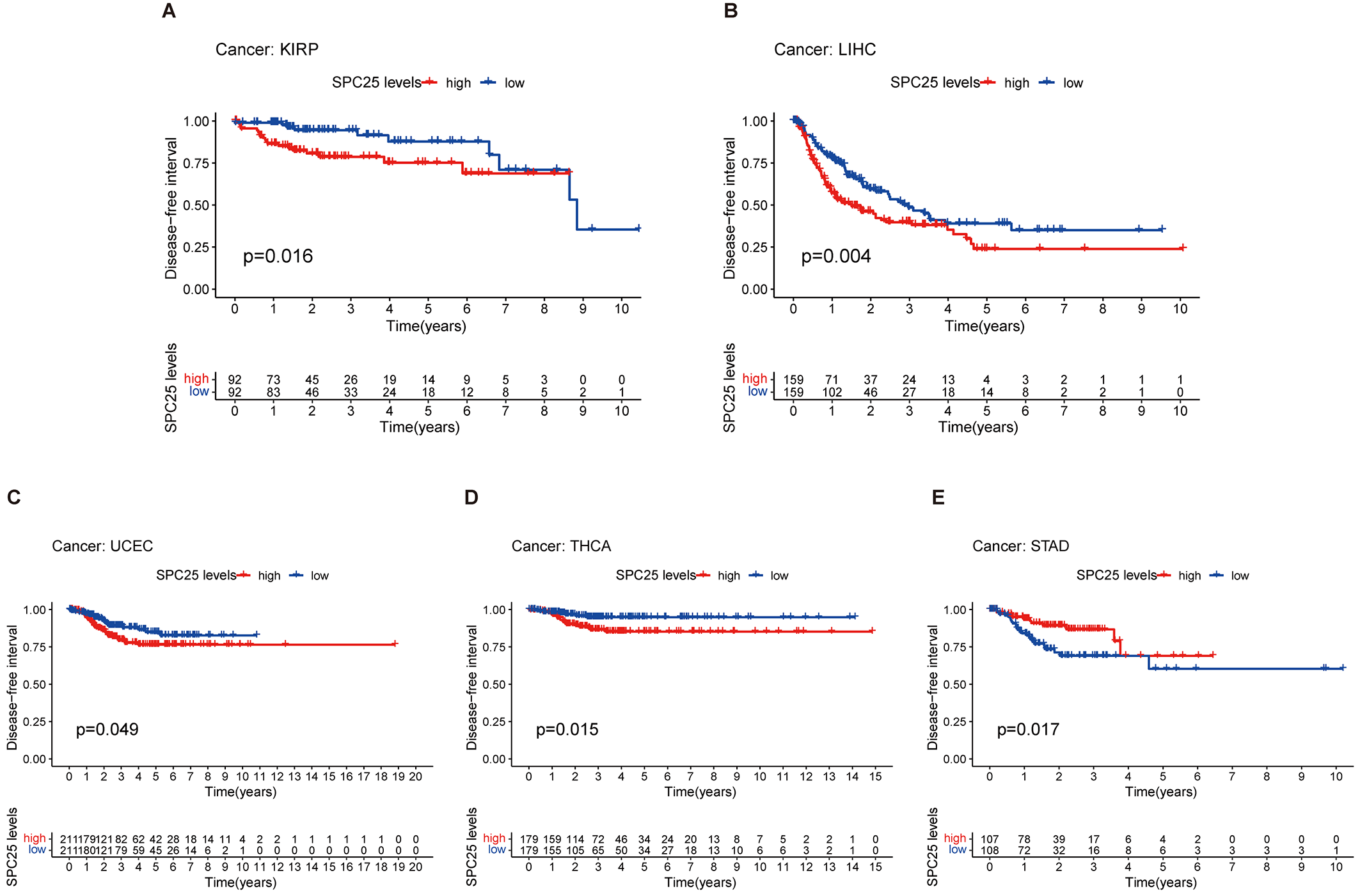

Supplement: Supplementary file 3 — Supplementary Figure 2. [file 41598_2024_59038_MOESM3_ESM.tif]

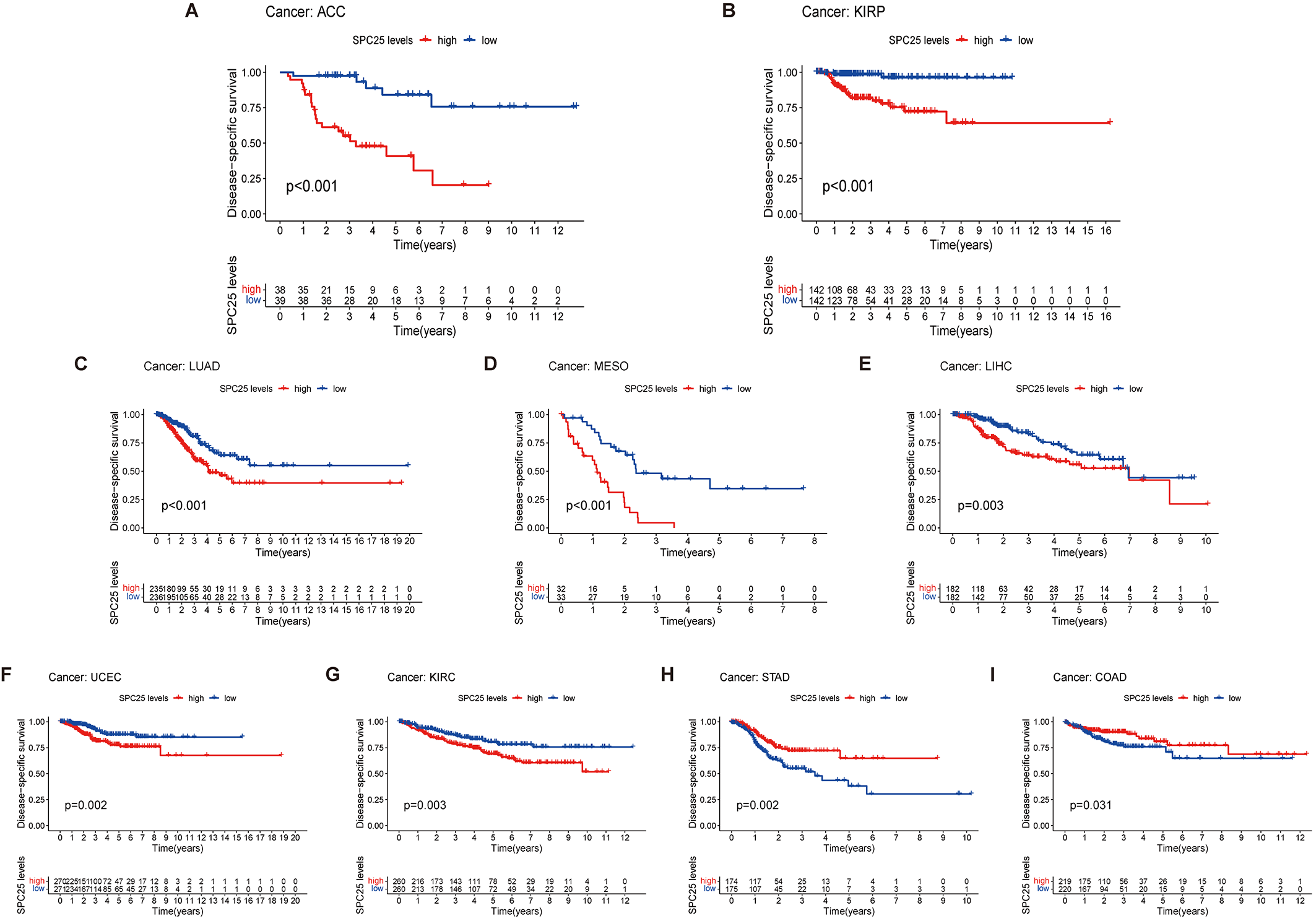

Supplement: Supplementary file 4 — Supplementary Figure 3. [file 41598_2024_59038_MOESM4_ESM.tif]

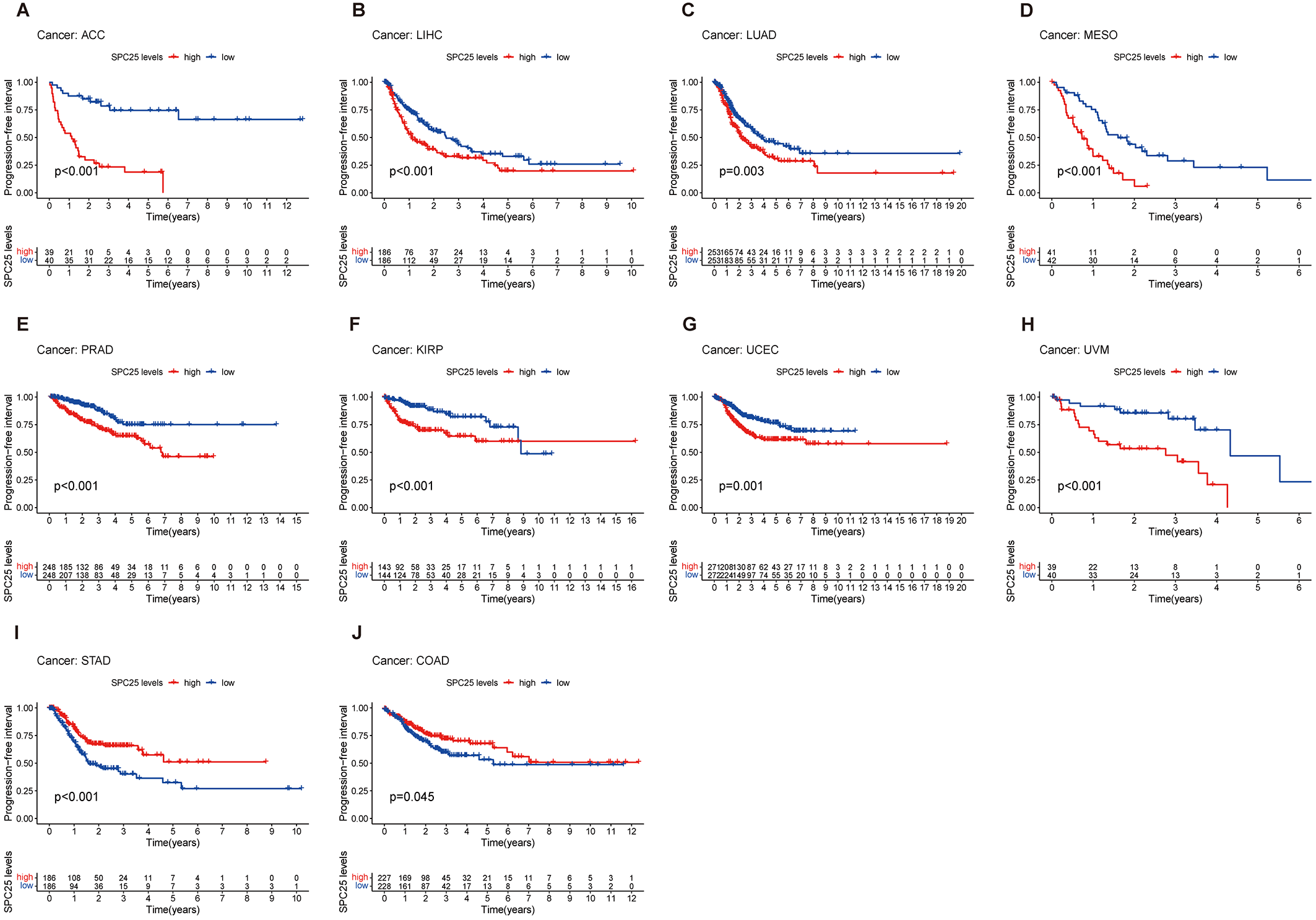

Supplement: Supplementary file 5 — Supplementary Figure 4. [file 41598_2024_59038_MOESM5_ESM.tif]

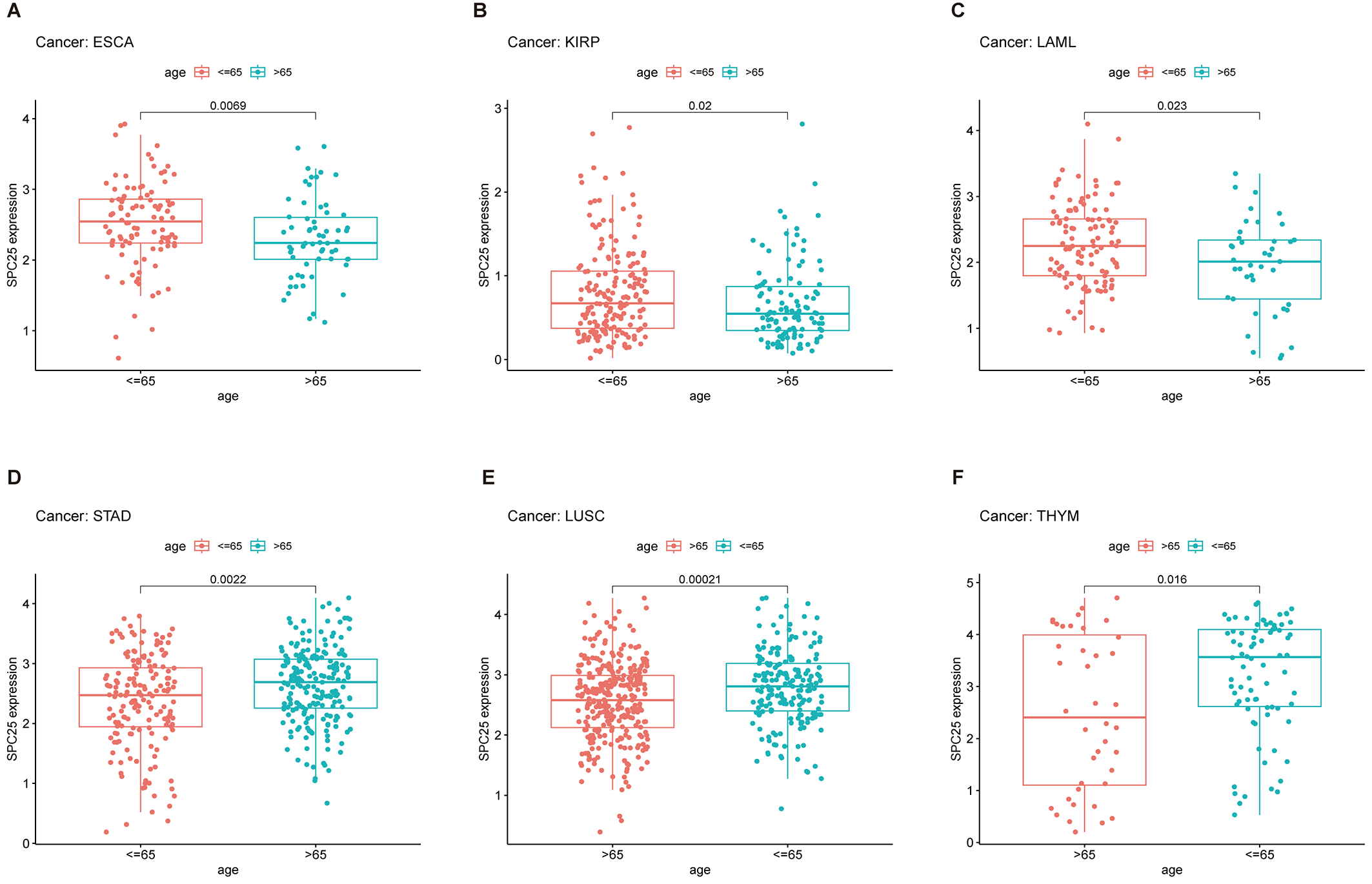

Supplement: Supplementary file 6 — Supplementary Figure 5. [file 41598_2024_59038_MOESM6_ESM.tif]

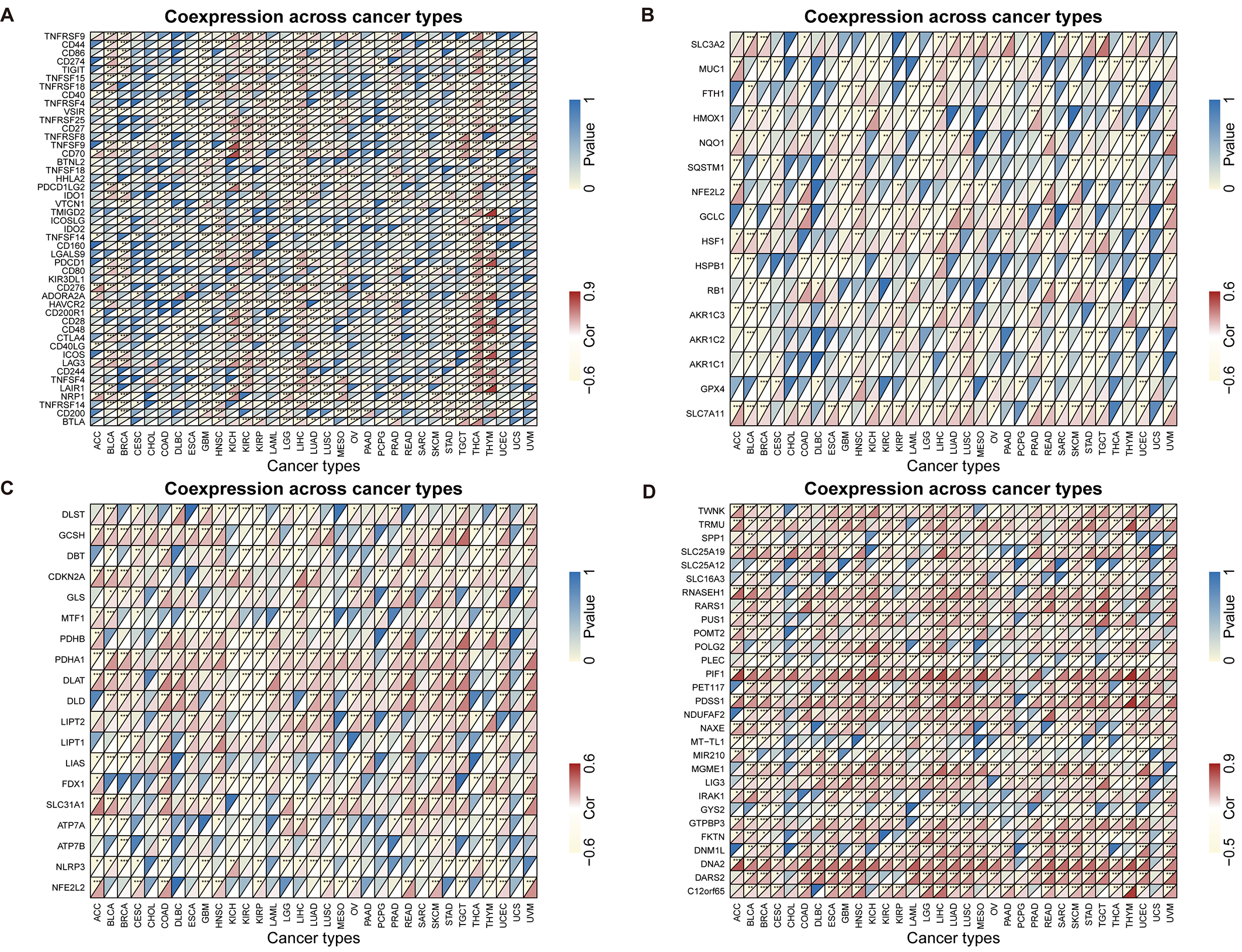

Supplement: Supplementary file 7 — Supplementary Figure 6. [file 41598_2024_59038_MOESM7_ESM.tif]
